# An optimized method for plasma extracellular vesicles isolation to exclude the copresence of biological drugs and plasma proteins which impairs their biological characterization

**DOI:** 10.1371/journal.pone.0236508

**Published:** 2020-07-29

**Authors:** Onno J. Arntz, Bartijn C. H. Pieters, Peter L. E. M. van Lent, Marije I. Koenders, Peter M. van der Kraan, Fons A. J. van de Loo

**Affiliations:** Department of Rheumatology, Radboud University Medical Center, Nijmegen, The Netherlands; Emory University School of Medicine, UNITED STATES

## Abstract

Extracellular vesicles (EVs) are cell membrane-derived phospholipid bilayer nanostructures that contain bioactive proteins, enzymes, lipids and polymers of nucleotides. They play a role in intercellular communication and are present in body fluids. EVs can be isolated by methods like ultracentrifugation (UC), polyethylene-glycol-precipitation (PEG) or size exclusion chromatography (SEC). The co-presence of immunoglobulins (Ig) in EV samples isolated from plasma (pEVs) is often reported and this may influence the assessment of the biological function and phenotype of EVs in bio- and immunoassay. Here, we studied the presence of an Ig-based therapeutic (etanercept) in pEV samples isolated from rheumatoid arthritis (RA) patients and improved the isolation method to obtain purer pEVs. From plasma of etanercept (Tumor-necrosis-factor (TNF)-α antibodies)-treated RA patients pEVs were isolated by either UC, PEG or SEC. SEC isolated pEVs showed the highest particle-to-protein ratio. Strong TNF-α inhibition determined in a TNF-α sensitive reporter assay was observed by pEVs isolated by UC and PEG, and to a lesser extent by SEC, suggesting the presence of functional etanercept. SEC isolation of etanercept or labelled immunoglobulin G (IgG) showed co-isolation of these antibodies in the pEV fraction in the presence of plasma or a high protein (albumin) concentration. To minimize the presence of etanercept or immunoglobulins, we extended SEC (eSEC) column length from 56mm to 222mm (total stacking volume unchanged). No effect on the amount of isolated pEVs was observed while protein and IgG content were markedly reduced (90%). Next, from six etanercept- treated RA patients, pEVs were isolated on a eSEC or standard SEC column, in parallel. TNF-α inhibition was again observed in pEVs isolated by conventional SEC but not by eSEC. To confirm the purer pEVs isolated by eSEC the basal IL-8 promoter activation in human monocytes was determined and in 4 out of 5 SEC isolated pEVs activation was observed while eSEC isolated pEVs did not. This study shows that extended SEC columns yielded pEVs without detectable biologicals and with low protein and IgG levels. This isolation method will improve the characterization of pEVs as potential biomarkers and mediators of disease.

## Background

Extracellular vesicles (EVs) are a heterogeneous group of membrane-covered nanoparticles of diverse sizes and shapes produced by almost all cells. There are three different types of EVs: exosomes (30-100nm), microvesicles (100-1000nm) and apoptotic bodies (1–10μm) which are classified based on their origin. Apoptotic bodies originate from apoptotic cells, microvesicles from the plasma membrane and exosomes from multivesicular bodies [1–3]. EVs can be found in all body fluids such as breast milk, saliva, urine and blood [4] and their lipid bilayer membrane protect their content from proteases and nucleases present in body fluids. EV content consist out of different proteins, lipids, RNA and DNA molecules [5] and their role as important cell-to-cell communicators is widely accepted [6]. For instance transfer of proteins and/or RNA by EVs can affect gene expression in acceptor cells including genes that are involved in auto-immune diseases [7] and inflammatory processes [8].

To investigate the functional role of plasma EVs (pEVs) accurately the purity of pEVs is highly important. The presence of immunoglobulins (Ig) in pEV samples is a general finding using ultracentrifugation (UC), polyethylene glycol precipitation (PEG) or size exclusion chromatography (SEC) as isolation methods, but is likely a co-isolated protein contaminating pEVs [9]. UC is based on sedimentation of solutes including EVs at a high centrifugation force. This isolation method might lead to aggregation and co-precipitation with soluble proteins present in the biofluid, such as albumin in plasma. It can even cause vesicle rupture or fusion with contaminants and other proteins, affecting the physical properties of the EVs and downstream analysis of the preparation [10–12]. PEG is an easy and cheap method to obtain the highest EV recovery. EV containing fluids are incubated with PEG and after centrifugation EVs are pelleted. PEG isolated EVs are commonly contaminated with protein aggregates and the precipitating agent is still present [13, 14]. SEC is based on the differential elution profiles of particles of different sizes running through a porous polymer, constituting the stationary phase—also known as gel filtration matrix or resin—and carried through the mobile phase of the SEC column. EVs, being bigger than the polymer’s pores, travel quicker along the column and therefore elute first, right after the column’s void volume [15]. While SEC allows the separation of EVs from HDL particles found in plasma, it cannot fully exclude other lipoproteins like chylomicrons (75–1200 nm) or VLDL (30–80 nm) [16].

The presence of therapeutic biologicals (Ig’s) in isolated pEV samples is as far as we know never been studied. Therefore pEVs were isolated by UC, PEG and SEC from plasma obtained from etanercept-treated rheumatoid arthritis (RA) patients. Optimization of SEC resolution by changing column length, column diameter, and loading volume suggested by by Boïng *et al*. [17] was also investigated by creating an extended four times longer SEC column (eSEC) containing the same stacking volume. Presence of etanercept and levels of IgG were measured in pEV samples and an optimized isolation method for EVs out of plasma is claimed.

## Material and methods

### Blood donors

Blood was obtained from 15 etanercept (50mg 1x per two week subcutaneous)-treated RA patients fulfilling ACR/EULAR classification [18] with active RA with DAS28 > 3.2 and at least one clinically painful and swollen joint. Patients were recruited at the outpatient clinic of the Department of Rheumatic Diseases of the Radboudumc and blood was donated between January 2016 and December 2018. The study was approved by the Institutional Review Board (IRB) of the Radboudumc (CMO region Arnhem-Nijmegen; dossier 2015–1847) and conducted according to the Declaration of Helsinki. The study protocol inclusion was limited to patients undergoing venapuncture for regular clinical care. Verbal explanation of the nature and scope of the research project was obligated, according to the protocol and applicable Dutch laws. Oral informed consent was obtained after explanation of the study. Documentation of consent in the electronic patient records to the participation in the specific protocol was considered sufficient and approved by the IRB. After consent an extra blood sample was taken in these patients undergoing venapuncture for regular clinical care. Study material was directly anonymized after withdrawal. Age, gender, presence of rheumatoid factor and time of using etanercept of each decoded donor is mentioned in supplement (S1 Fig). The effect of treatment on clinical parameters (Disease activity score, DAS; Erythocyte sedimentation rate, ESR; C-reactive protein, CRP; Visual analogue scale, VAS; Tender joint count; TJC; Swollen joint count; SJC) obtained by physicians before and after etanercept treatment were calculated as delta (Δ).

### Blood sample preparation

Blood samples were taken in ethylenediaminetetraacetic acid tubes (BD, Plymouth, United Kingdom) and within 1 hour centrifuged for 10 minutes at 1690g by 4°C to obtain plasma. This plasma was centrifuged at 10,000g for 30 minutes at 4°C to obtain platelet free plasma (pfp). Thereafter it was passed through a 0.22μm filter (Whatmann, GE Healthcare, Buckinghamshire, United Kingdom) and aliquoted. Aliquots were stored at -80°C

### Plasma EV isolation

#### UC

To 500μl pfp 3500μl phosphate buffered saline (PBS) was added. After 2 hrs centrifugation 110,000g at 4°C in a SW60 rotor (Beckman Coulter, Woerden, The Netherlands) pellet was resuspended in 500μl PBS and stored at 4°C till for further use.

#### PEG precipitation

To 500μl pfp 100μl 50% PEG6000 (Sigma-Aldrich, St. Louis, MO, USA) was added and after overnight incubation at 4°C sample was centrifugated at 1500g for 30 minutes. Pellet was resuspended in 500μl PBS and stored at 4°C for further use.

#### SEC

pEVs were isolated by SEC using the protocol described by Lobb *et al*. [13]. In short, a sterile column was prepared for SEC using a 10 ml syringe stacked with sepharose CL-2B (Pharmacia, Uppsala, Sweden). After washing the column with PBS containing 0.32% citrate (pH 7.4, autoclaved), 500μl of pfp was loaded and eluted using PBS/0.32% citrate buffer. All 1 ml eluate fractions were collected and stored at 4°C for further use.

#### eSEC

To optimize the resolution capacity of SEC isolation method the column was elongated following the recommendations from Boïng *et al* [17]. The height of the stacked column was increased from 56 mm (10ml syringe) to 222 mm by using a 10ml serological pipette (Greiner Bio-One, Frickenhausen, Germany) with a shrinked diameter. The total volume (10ml) of stacking material (Sepharose CL-2B) was equal to the conventional SEC column. pEVs were isolated as previous described and all 1 ml eluate fractions were collected and stored at 4°C till further analysis was performed.

### Protein measurement

The amount of protein was measured using a Micro BCA Protein assay kit following the protocol provided by the manufacturer (Thermoscientific, Rockford, USA). pEV samples were diluted 10, 20, 40 and 80 times in NaCl 0.9% and after 2h incubation at 37°C absorbance was measured using the BioRad iMark microplate reader. Concentration was calculated by using the absorbance of a standard curve of albumin (BSA).

#### Nanoparticle Tracking Analysis

Vesicle size distribution was estimated by the Brownian motion of particles using a NanoSight NS300 (Sysmex, Etten-Leur, The Netherlands) with Nanoparticle Tracking Analysis 3.2 software (NanoSight, Amesbury, United Kingdom). Vesicles were diluted in PBS till an optimal concentration for reliable analysis was reached (20–80 particles per frame). Each sample was measured for 60 seconds, using the following software settings: flow rate 50, camera level 10 and detection threshold 5.

### TNF-α sensitive reporter assay

THP1 cells with an IL-8 promoter driven-firefly luciferase expression construct were used to measure TNF-α inhibition of the obtained pEV samples. 50,000 cells per well were plated out in a 96-wells flat bottom plate. 12,5μl of each pEV sample with a suboptimal dose TNF-α (12.5μl of 250pg/ml) was added and after 6 hr cultured by 37°C with 5% CO_2_ the plate was centrifugated for 5 min (1700RPM) and supernatants were discarded. Cells in pellet were lysed with 30μl milliQ and firefly luciferase was measured by adding 20μl Bright Glo (Promega, Leiden, The Netherlands) in a luminometer (Clariostar BMG, Isogen, De Meern, The Netherlands). TNF-α inhibition was calculated in percentage with TNF-α (12.5μl, 250pg/ml) plus 12.5μl PBS as negative control (0%) and only pbs without TNF-α as positive control (100%). To detect the direct effect of pEVs to the inducible IL-8 promotor the same protocol was used except no TNF-α was added.

### IgG detection

IgG levels in pEV samples were determined by ELISA. In short, 0.2μg polyclonal Goat-anti-humIgG(H+L) (Jackson Immuno Research, Cambridgeshire, United Kingdom) was coated in a 96 wells flat bottom plate. After washing with PBS+0.05%Tween-20 (Millipore), pEVs were added and incubated for 90 min at RT. Wells were washed twice with PBS-Tween and polyclonal Goat-anti-humIgG(Fab)ALEXA488 (Bioconnect, Jackson, Huissen, The Netherlands) was added and incubated for 90 min at RT. Following a third washing step amount of ALEXA488 was determined in a fluorescence meter (Clariostar BMG). IgG concentrations were calculated using a standard curve of human IgG (Sigma-Aldrich, St. Louis, MO, USA).

### IgG-ALEXA488 detection

To detect SEC elution of IgG Alexa488 labelled hum-IgG1 (Bio techne Ltd, Abington, United Kingdom) was used. After 10 min preincubation in the presence of pfp or pbs 1μg/ml Alexa488 labelled hum-IgG1 was eluted and in each 1 ml fraction Alexa488 was determined in a fluorescence meter (Clariostar BMG) as relative fluorescence units (RFU). Amounts of Ig were showed in graphs as ΔRFU (RFU of Alexa488 labelled hum-IgG1 minus RFU of pbs]

### Statistical analysis

All data are expressed as mean±SD. Data were compared using two tailed Mann-Whitney U-test, Two-way ANOVA with Bonferroni’s post hoc test or paired T-test as mentioned in figures. Values of *P*<0.05 were considered to indicate statistical significance. All statistical analyses were performed using GraphPad Prism 5.01 (GraphPad Software, La Jolla, CA).

## Results

### TNF-α inhibition in SEC isolated pEVs of RA patients treated with etanercept

**Fig 1:**
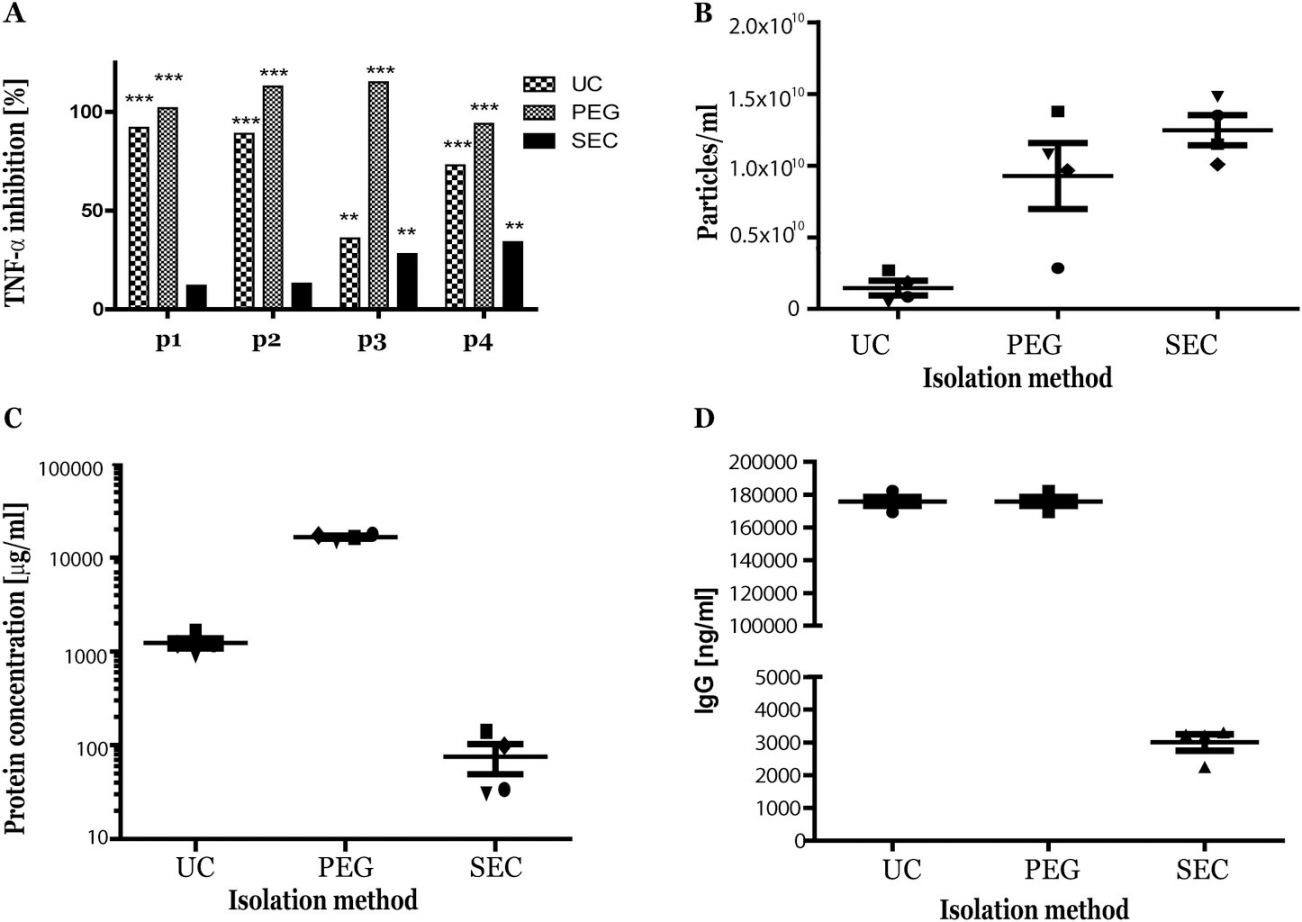
Presence of etanercept in pEV samples isolated from RA patients. From 4 etanercept-treated RA patients pEVs were isolated by three different conventional isolation methods (UC, PEG or SEC). TNF-α inbition was determined in all obtained pEV samples using a TNF-α sensitive reporter assay (A). Next, particle and protein concentration was measured by Nanosight tracking analysis (NTA) (B) and micro-BCA protein assay (C). Own IgG levels in pEV samples were measured by ELISA (D). Statistically significant TNF-α reduction versus control (only TNF-α without pEVs) was determined by Mann-Whitney test, ** p<0.01, *** p<0.001.

From four etanercept-treated RA patients (p1-2-3-4) pEVs were isolated by three conventional isolation methods (UC, PEG or SEC) in parallel. To detect the presence of etanercept in isolated pEV samples a TNF-α sensitive assay was used. After preincubation of the pEV samples with a suboptimal dose of TNF-α, a significant TNF-α inhibition was observed with pEVs isolated by UC or PEG and to a lesser extent by SEC, suggesting the presence of etanercept in isolated pEV samples (Fig 1A). Next, the isolated pEVs were characterized and plasma EVs obtained by SEC isolation showed the highest number of particles (Fig 1B) with the lowest protein levels (Fig 1C). In the isolated pEV samples the presence of patients owns IgG levels were detected by ELISA and also these levels were markedly lower in SEC isolated pEVs (Fig 1D).

### Etanercept is co-isolated with pEVs by SEC in the presence of plasma

**Fig 2:**
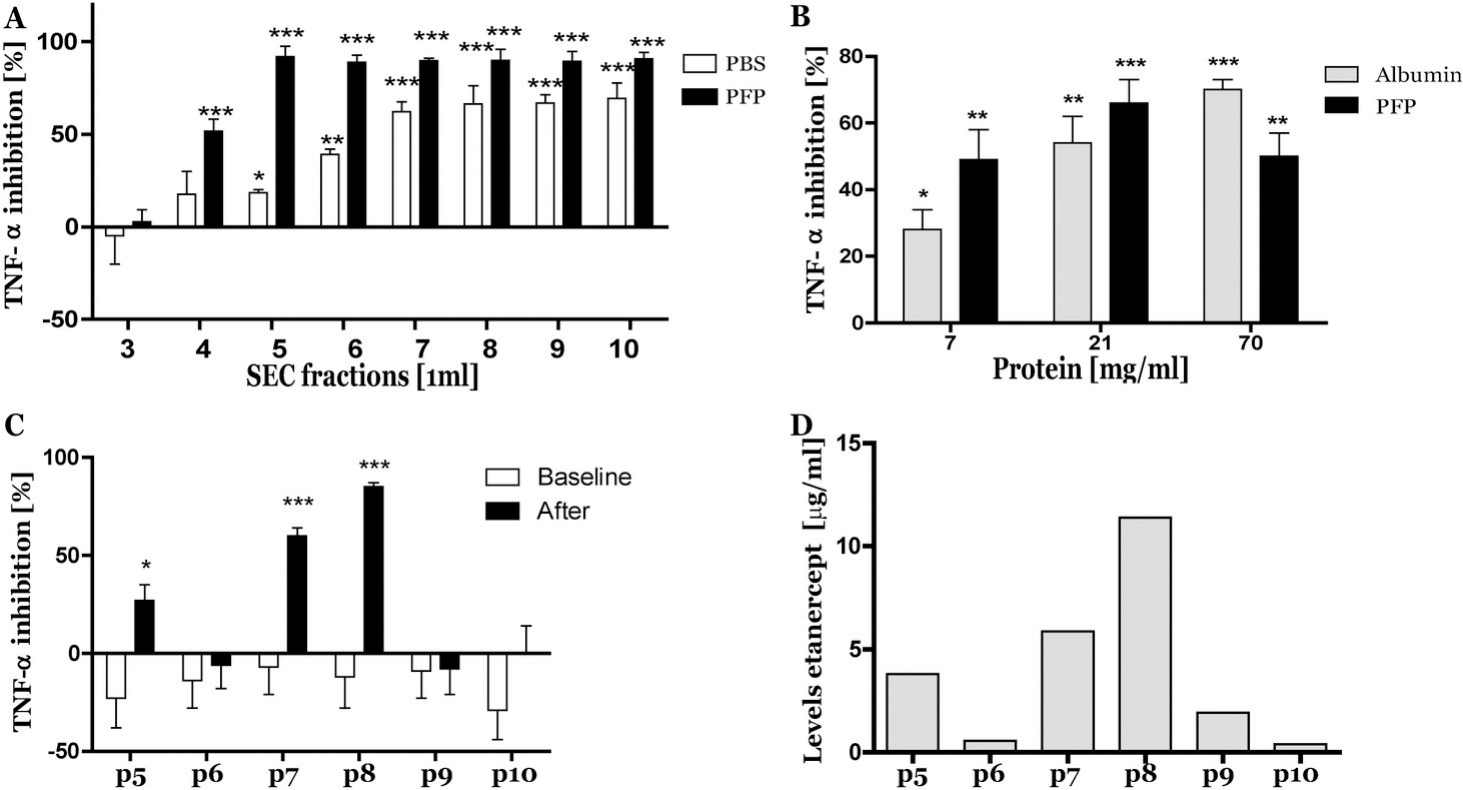
Presence of plasma proteins leads to co-isolation of etanercept in the SEC pEV fraction. Etanercept (1μg/ml) in PBS alone or with pfp was SEC isolated. In each eluted SEC fraction, TNF-α inhibition was measured in triplo (A). Etanercept (1μg/ml) in the presence of 3 different concentrations pfp (10-20-100%) or equal protein levels of albumin (7-21-70 mg/ml) was SEC isolated. In Fraction 5 (pEV fraction) TNF-α inhibition was measured in triplo (B). Next, levels of etanercept were measured in plasma of 6 new etanercept treated RA patients (C). Finally, in these RA patients neutralization of TNF-α was measured in triplo on SEC isolated pEVs before (white bars) and after (black bars) etanercept treatment (C). Statistically significant differences in TNF-α reduction versus control (only TNF-α without pEVs), were determined by Mann-Whitney test, * p<0.05, ** p<0.01, *** p<0.001.

The observed presence of etanercept in pEV samples could be pEV bound or could be a co-isolation artefact caused by binding of etanercept to plasma proteins. To elucidate the presence of etanercept in the pEV fraction, a therapeutically relevant observed concentration of etanercept (1ug/ml) [19] was spiked to 500μl human pfp (healthy control) or 500μl PBS. TNF-α inhibition was determined in all SEC elution fractions. Without plasma, a marginal TNF-α inhibition was observed in the first eluted fraction after void volume (fraction 4) and a maximum inhibition (62%) in fractions 7 up to 10. In the presence of platelet free plasma (pfp) a significant TNF-α inhibition (52%) was already observed in SEC fraction 4, and from fractions 5 till 10 an inhibition of 90% was observed (Fig 2A). This experiment was repeated with another healthy plasma donor and equal results were obtained (data not shown). Next, etanercept (1ug/ml) was incubated with different volumes pfp (50, 150 and 500μl) or as control corresponding protein (albumin) concentrations (7, 21, 70 mg/ml). Most EVs were eluted in fraction 5 and in this fraction TNF-α inhibition was determined. With the lowest amount of added albumin (7 mg/ml) 28% TNF-α inhibition was observed which increased to 50–70% with higher amounts of albumin while each added pfp volume showed a strong TNF-α inhibition (Fig 2B). To investigate TNF-α inhibition by the presence of etanercept, from six new untreated RA patients (p5-6-7-8-9-10) pEVs were isolated by SEC at baseline (before start of etanercept administration) and after treatment. No TNF-α inhibition was observed at baseline in the isolated pEVs, but after their first etanercept administration TNF-α inhibition was observed in 3 out of 6 (Fig 2C). Etanercept levels in pfp of these RA patients were measured by the TNF-α inhibition assay with a dose response curve of known levels of etanercept. Observed etanercept levels were between 0.4 and 11.4 μg/ml (Fig 2D). Particle concentration and protein content of all isolated RA pEV samples showed no significant differences and were also not statistical different to pEVs isolated from healthy controls (n = 5) (S2 Fig).

### IgG in the presence of plasma is eluted earlier from SEC column

**Fig 3:**
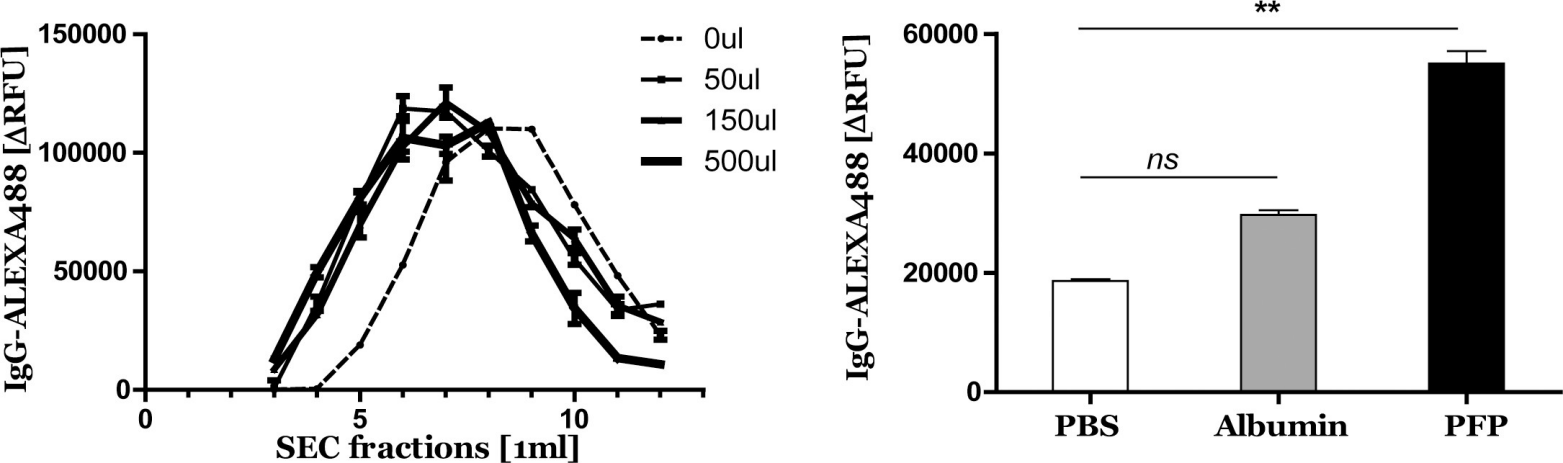
Presence of plasma proteins leads to co-isolation of IgG in the SEC pEV fraction. Alexa488 labelled IgG (1μg/ml) was SEC isolated with three different pfp concentrations or PBS. Alexa488 was measured in each eluted SEC fraction by fluorometry (A). Alexa488 labelled IgG (1μg/ml) in the presence of pfp, equal protein level albumin (70mg/ml) or PBS was SEC isolated and Alexa488 was measured by fluorometry (B). Two-way ANOVA with Bonferroni’s post hoc test was used to compare PBS vs. additives, ** p<0.01.

To determine if the observed shift of etanercept in the presence of plasma proteins is a specific trait for etanercept or also holds true for other immunoglobulins, Alexa488-labelled IgG was incubated with different volumes of pfp (50, 150 and 500μl) and in each SEC fraction Alexa488 was measured by fluorometry. Labelled IgG in PBS started to elute in SEC fraction 5 with a peak of elution between fractions 8 and 9, while in the presence of pfp IgG was already detectable in fraction 4 and peak of elution was between fractions 6 and 7, independent of added pfp volume (Fig 3A). In the presence of albumin (70mg/ml) detectable IgG was higher in SEC fraction 5 than compared to PBS without albumin, but significant lower than in the presence of pfp (Fig 3B) suggesting some binding interaction of IgG to pfp proteins.

### pEVs obtained by longer SEC columns shows less protein impurities

**Fig 4:**
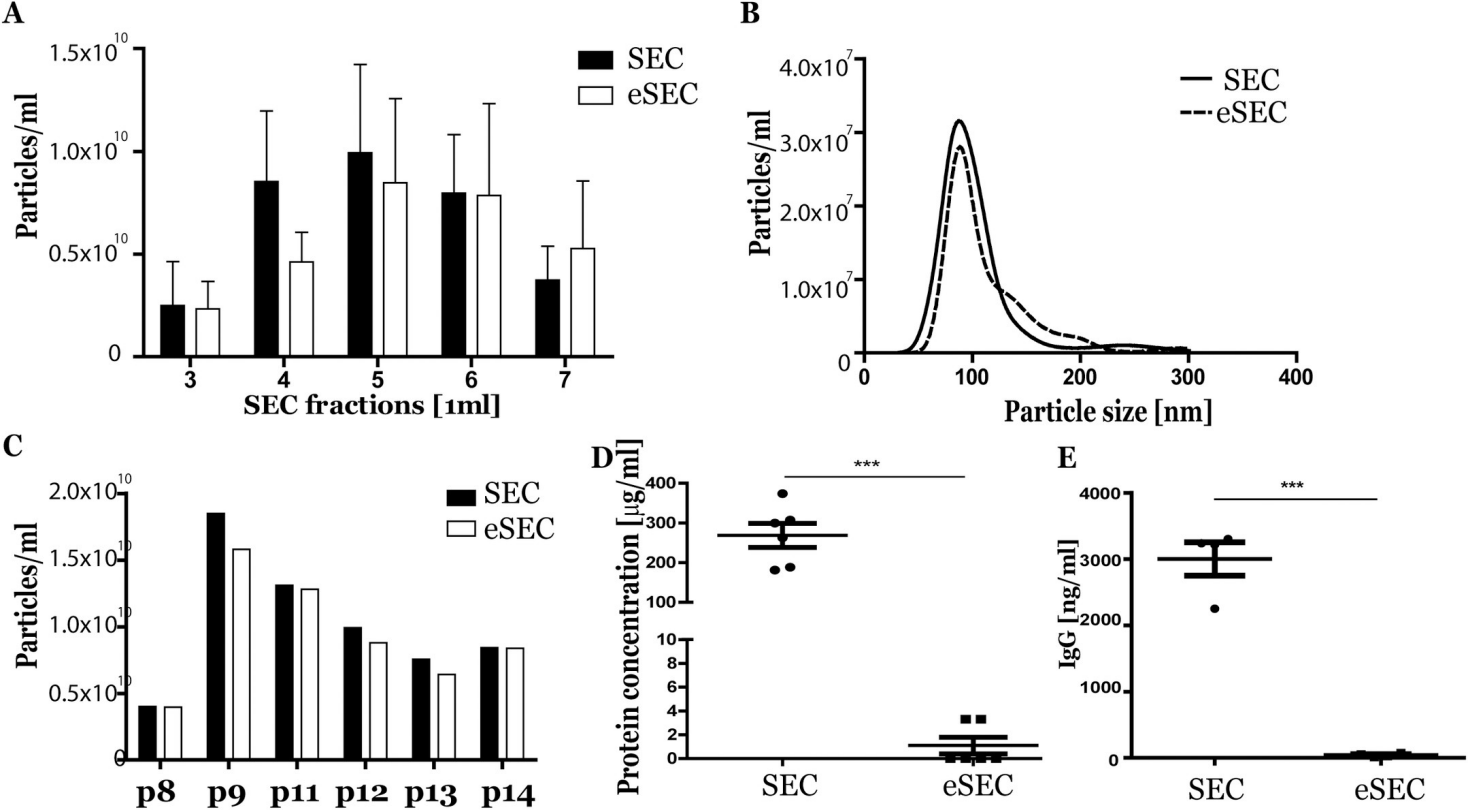
pEV isolation by extended SEC column shows less protein impurities. From 500μl pfp obtained from 6 RA patients pEVs were isolated by SEC or eSEC and particle concentration was measured in eluted fractions 3–7 by NTA (A). Compared SEC and eSEC size-profile from 1 representative pEV sample (fraction 5) is shown (B). Particle concentration of SEC or eSEC isolate pEVs in fraction 5 (n = 6) were measured by NTA (C). Protein content was measured by micro-BCA protein assay of each SEC or eSEC isolated pEV sample (D) and own IgG levels were measured by ELISA (E). Statistically significant differences in protein content were determined by paired T-test, *** p<0.001.

The isolation method was optimized to explicitly investigate the presence of etanercept on pEVs. From plasma donated by six etanercept-treated RA patients (p8-9-11-12-13-14) pEVs were isolated by conventional SEC or extended SEC (eSEC) columns in parallel. Obtained elution fractions were analysed and the highest amount of particles were observed in fraction 5 both for SEC and eSEC columns (Fig 4A). Also the pEV particle size distribution detected by NTA was not different, a representative size-profile is shown in Fig 4B. Particle concentration of SEC- or eSEC-isolated pEVs was not different for these six individual RA patients (Fig 4C), while protein levels determined by microBCA were highly significantly reduced in pEVs obtained by eSEC (Fig 4D). Also the levels of IgG detected by ELISA were markedly lower in eSEC isolated pEVs (Fig 4E).

### No TNF-α inhibition and IL8 promoter activation by eSEC isolated pEVs

**Fig 5:**
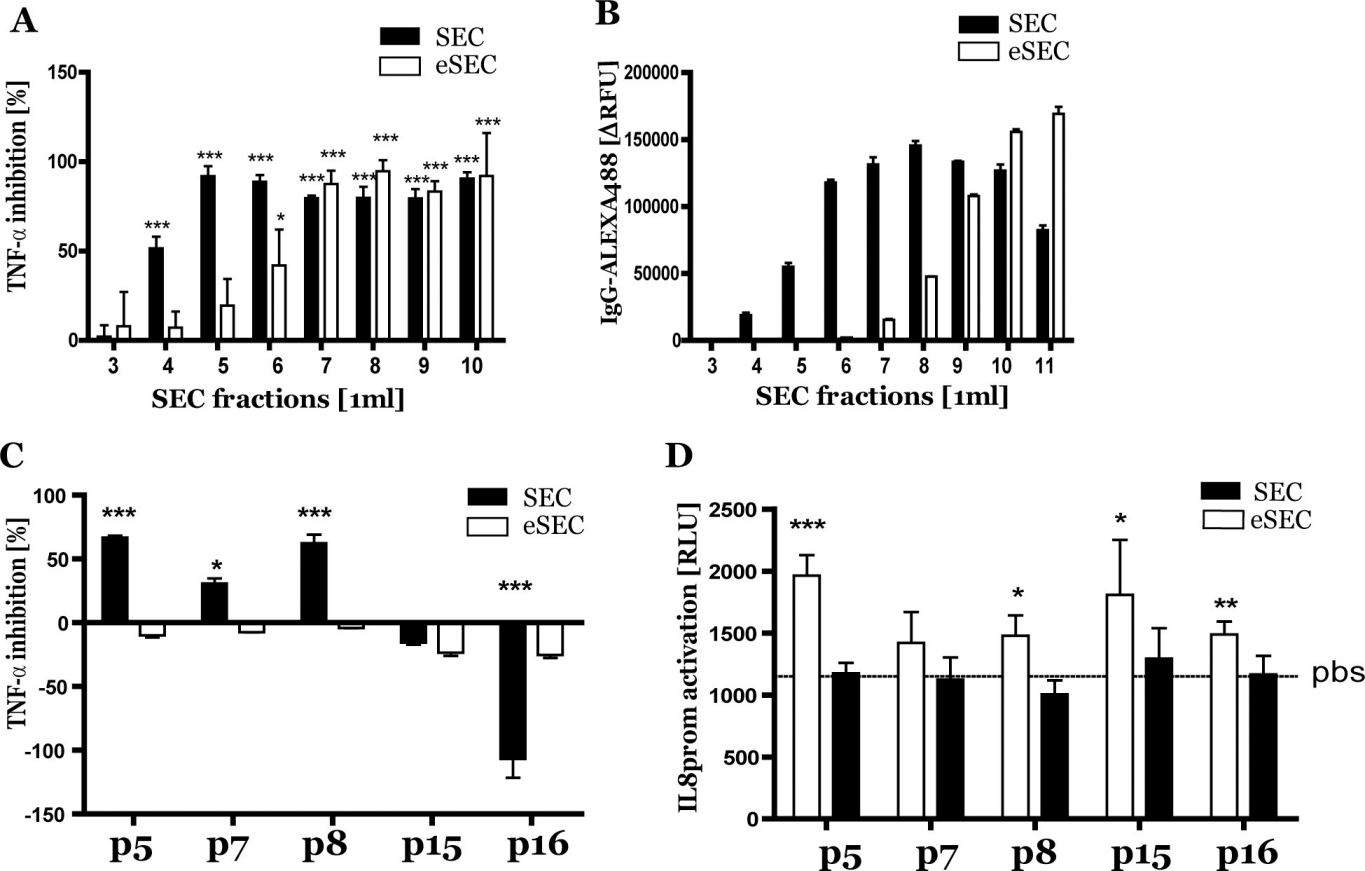
No TNF-α inhibition and IL8 promoter activation by eSEC isolated pEVs. Comparison of SEC vs eSEC isolation. Etanercept (1μg/ml) in the presence of 500μl pfp was isolated and TNF-α inhibition of each eluted SEC (black bars) or eSEC (white bars) fraction was determined using a TNF-α sensitive reporter bioassay (A). Also Alexa488 labelled IgG (1μg/ml) was SEC (black bars) or eSEC (white bars) isolated and in each fraction Alexa488 was measured by fluorometry (B). From 5 RA patients administered with etanercept pEVs were isolated by SEC (black bars) or eSEC (white bars) and TNF-α inhibition was determined (C). Direct biological effects of these isolated pEVs were studied in THP1 cells with an IL-8 promoter driven-firefly luciferase expression construct (D). Statistically significant differences were determined by Mann-Whitney test versus control (for A and D only TNF-α without pEVs and for E only pbs) or by paired T-test (C), * p<0.05, ** p<0.01, *** p<0.001.

Next we determined whether eSEC not only diminished the copurification of proteins in the pEV fraction, but also that of etanercept. Firstly, TNF-α inhibition of eSEC isolated etanercept (1μg/ml) in the presence of 500μl healthy control pfp was determined and compared to conventional SEC isolation. In fraction 5 no significant inhibition was observed with eSEC while by standard SEC isolation TNF-α inhibition was still observed (Fig 5A). Secondly, Alexa488 labelled IgG (1ug/ml) was isolated by eSEC or SEC in the presence of 500μl healthy control pfp. IgG was still detectable in fraction 5 by SEC isolation with a peak at fraction 8 while by eSEC isolation minimal IgG levels were observed in fraction 5 and maximal at fraction 11 (Fig 5B). Next, the presence of etanercept was determined in the SEC or eSEC isolated pEVs obtained from five RA patients. In SEC isolated pEVs TNF-α inhibition was observed in 3 out of 5 while 1 patient showed extra IL8 promotor activation. In the pEVs isolated by eSEC no TNF-α inhibition was observed suggesting no presence of etanercept (Fig 5D). Finally, the basal effect of these SEC or eSEC isolated pEVs on IL8-promotor activation in THP-1 cells was determined. IL8-promotor activation was observed in 4 out of 5 SEC pEVs samples while eSEC isolated pEVs showed no effect which confirm purer pEVs obtained by eSEC (Fig 5E).

## Discussion

This study investigated the necessity for an improved isolation method to obtain more pure pEVs out of blood. A four times extension of the SEC column with equal volume of stacking material, markedly reduced the protein and IgG levels, and the co-isolation of administered IgG-based biological was prevented. The observed biological effects of conventional isolated pEVs in this study were not found by our optimized isolation for purer pEVs.

We found that etanercept was detectable in some pEV samples isolated with generally accepted conventional methods from RA patients who were administered with this biological.

Etanercept can be detected in blood [19] of treated patients as we also showed in this study, but the presence in pEV samples was surprising. Not in all RA patients who received etanercept (one injection every two weeks) an inhibition of TNF-α was observed by the isolated pEV samples. The observed levels seems to correlated with the plasma levels. The different levels of etanercept could due to the difference in time between blood draw and administration of etanercept but unfortunately this information was not available. The technology used in the 3 isolation techniques is different. By UC and PEG, pEVs were pelleted and by SEC pEVs were obtained in solution. By SEC proteins smaller that 70nm (pore size of Sepharose CL-2B) moves slower through the column and will be separated from pEVs (around 100nm) while by UC and PEG the proteins probably will precipitated. Therefore SEC isolated pEVs are more pure as seen by NTA analysis (S3 Fig). SEC isolation of etanercept protein alone showed marginal TNF-α inhibition in the fraction immediately after the void volume collect, as expected. In contrast, in the presence of even the smallest amount of added plasma TNF-α inhibition was observed in all SEC fractions eluted after void volume, including the pEV elution fractions. SEC isolation of different volumes of plasma supplemented with pbs to 500μl, showed corresponding protein levels and particles concentrations to the amount of eluted plasma (S4 Fig). This observed shift in elution suggests binding of etanercept to pEVs and/or plasma proteins probably due to the Fc-tail of etanercept and Fc-gamma-binding proteins circulating in plasma [9]. Because of the presence of a Fc-tail in other neutralizing antibodies like Adalimumab it can also occur with other biologicals [20]. Most biological antibodies carry a positive charge [21] and complex formation with plasma IgGs, which are negative charged under physiological conditions [22] could also lead that etanercept elutes off the SEC column faster than without plasma.

SEC isolation of etanercept protein in the presence of a low concentration of albumin (7 mg/ml) showed TNF-α inhibition in fraction 5 which was enhanced by adding higher albumin concentrations. Protein levels in plasma are around 70 mg/ml [23] and this suggested that protein-saturation of the SEC column is an obvious explanation for the presence of etanercept in SEC isolated pEV samples. Saturation by the amount of loaded EVs is not presumable because this isolation method is also general used for isolation of other EVs were high amounts of EVs are present [24]. These observations suggests that EV isolation out of plasma is not suitable by standard SEC isolation method. Therefore we optimized the SEC isolation following the recommendations from Boïng *et al*. [17] by extending the SEC column length four times using the same volume of stacking material as used in the standard SEC column length. The size distribution of pEVs isolated with the conventional or extended SEC column showed overlapping profiles and corresponded to previously described pEV sizes [25]. The extended column showed improved particle to protein separation which is in line with literature, which states that longer column lengths result in better resolution [26]. The isolated amounts of pEVs by SEC or eSEC were equal although protein levels were limited in the eSEC isolate pEVs. It is known that also protein aggregates can be measured by NTA [27]. The equal observed amounts of pEVs isolated by SEC or eSEC suggested that no or NTA undetectable protein aggregates were present in SEC isolated pEVs. The eSEC isolated pEVs were still CD63 positive (S5 Fig) and maintained the floating density described for EVs (S6 Fig) [28]. The great advantage of the extended SEC column is that the pEV eluted fractions were devoid of any TNFα-neutralizing effect suggesting no to limited copurification of etanercept that the patients were treated with.

With the extended column, the protein and IgG content were strongly reduced (< 90%) showing that eSEC isolated pEVs are more pure with low levels of IgG still detectable. RA is an autoimmune disease where B-cells plays an important role [29] and it is known that B-cells also release EVs that contain the B-cell receptor on their surface which is an immunoglobulin [30]. Therefore the presence of IgG+ pEVs isolated from plasma of RA patients can be explained. IgG+ pEVs could also come from primary leucocytes as earlier described by Saunderson *et al*. [31]. Which isotype of IgG and how it is presented by pEVs remains to be determined. One possibility is that IgGs are coupled to Fc binding proteins that are present on pEVs [32]. Another explanation is that IgG is bound to rheumatoid factor (RF-IgM) on pEVs as we have found in a subpopulation of RA patients [33]. An a-specific binding of etanercept to pEVs can be excluded because no TNF-α inhibition was observed in the EV fraction (fr 5) after eSEC isolation of HC- or RA- pEVs (isolated by eSEC) preincubated with etanercept (S7 Fig).

To obtain purer pEVs the obvious option is by immunoaffinity [34]. The heterogeneity of EV population and lack of a pan protein EV marker makes this a selective approach for subpopulations of EVs and furthermore the presences of IgG or Ab-based biologicals could further impair this method. The recent described Ion-exchange chromatography purification of EVs seems to be a good alternative to obtain pure pEVs. This method is used to isolate EVs from amniotic fluid [35] and cell cultured medium [36] while direct EV isolation out of plasma by this method is never been described so far and needs further investigation.

The observed higher purity of eSEC isolated pEVs isolated could be a step forward to investigate the biological function of pEVs or their use as novel biomarkers in arthritis–and possibly other autoimmune patients.

## Conclusion

The present study shows that conventional EV isolation methods used for human plasma leads to poor separation of EVs from large proteins such as immunoglobulins and Ig-based therapeutics, influencing the biological activity of pEVs. By extending the length (not volume) of the SEC column the retention of proteins increases resulting in more pure pEVs that will be more accurately allow immunophenotypic and the biologic characterization of pEVs from patients.

## Supporting information

S1 Fig Available information of each RA patient is shown. Delta of Disease activity score (DAS), erythroyte sedimentation rate (ESR), C-reactive protein (CRP), Visual analoge scale (VAS), tender joint count (TJC) and swollen joint count (SJC) were calculated (After treatment minus baseline).
(TIF)

S2 Fig Protein content and particle concentration of SEC isolated pEVs. Protein content and particle concentration of pEVs isolated from 6 RA patients before (white bars) and after (black bars) etanercept treatment and 5 age matched healthy controls (grey bar) detected by resp. micro-BCA (A) and NTA (B).
(TIF)

S3 Fig Distribution size of UC, PEG and SEC isolated pEVs. Distribution size of pEVs isolated by UC, PEG or SEC from the same RA patient. Most monodisperse particles were observed after SEC isolation.
(TIF)

S4 Fig Protein content of SEC isolated pEVs isolated from different volumes plasma. Protein content per particle of pEVs isolated by SEC of different pfp volumes (50-150-500μl) suplemented with pbs to 500μl. Enhanced protein per particle levels were observed with higher pfp volumes.
(TIF)

S5 Fig CD63 detection on SEC and eSEC isolated fractions. CD63 was detected in different SEC and eSEC isolated fractions. Highest CD63 content was observed in the pEV fraction (fraction 5).
(TIF)

S6 Fig Iodixanol density gradient of eSEC isolated pEVs. To detemined the density of the SEC and eSEC isolate pEVs a discontinuous iodixanol gradient was used and the most pEVs were detected in the 20% density layer.
(TIF)

S7 Fig No a-specific binding of etanercept to eSEC isolated pEVs. Detemination of TNF-α inhibition in eSEC isolated fractions of HC- or RA- pEVs (isolated by eSEC) preincubated with etanercept (1 μg/ml. No TNF-α inhibition was observed in eSEC fractions 4, 5 and 6.
(TIF)

S1 Data (DOCX)

## List of abbreviations

EV: Extracellular Vesicle
SEC: Size Exclusion Chromatography
UC: Ultracentrifugation
PEG: Polyethylene Glycol precipitation
Pfp: Platelet free plasma
RA: Rheumatoid Arthritis
IgG: Immunoglobulin G
TNF-α: Tumor necrosis factor α
NTA: Nanoparticle Tracking Analysis
