# Supplementary figures and images for: An optimized method for plasma extracellular vesicles isolation to exclude the copresence of biological drugs and plasma proteins which impairs their biological characterization
Source: PLoS One. 2020 Jul 29;15(7):e0236508. doi: 10.1371/journal.pone.0236508 (PMC7390383; doi:10.1371/journal.pone.0236508)

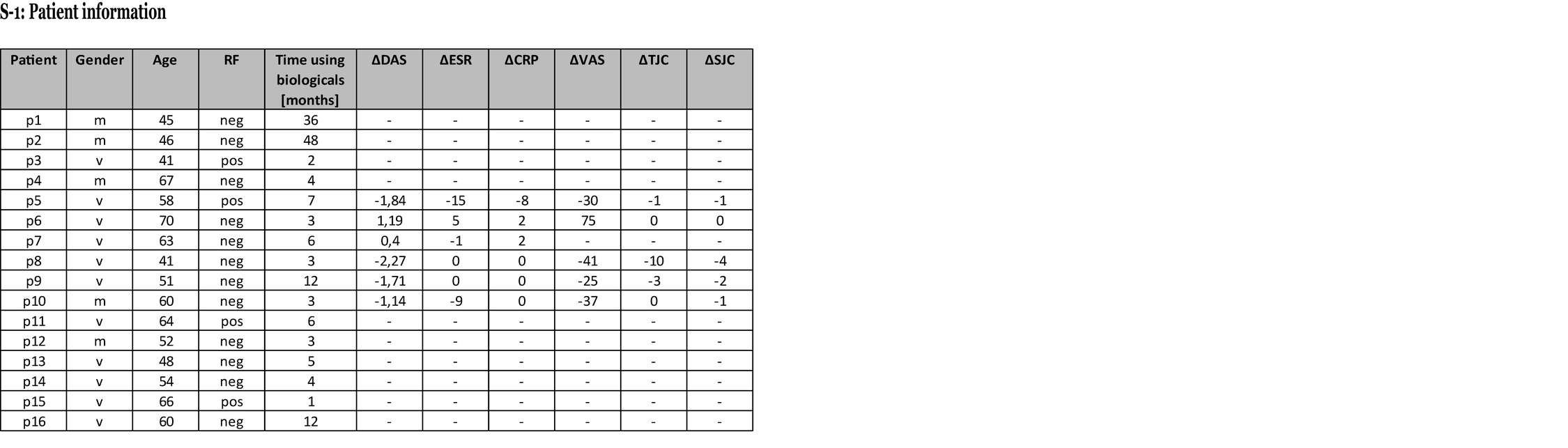

Supplement: S1 Fig — Delta of Disease activity score (DAS), erythroyte sedimentation rate (ESR), C-reactive protein (CRP), Visual analoge scale (VAS), tender joint count (TJC) and swollen joint count (SJC) were calculated (After treatment minus baseline). (TIF) [file pone.0236508.s001.tif]

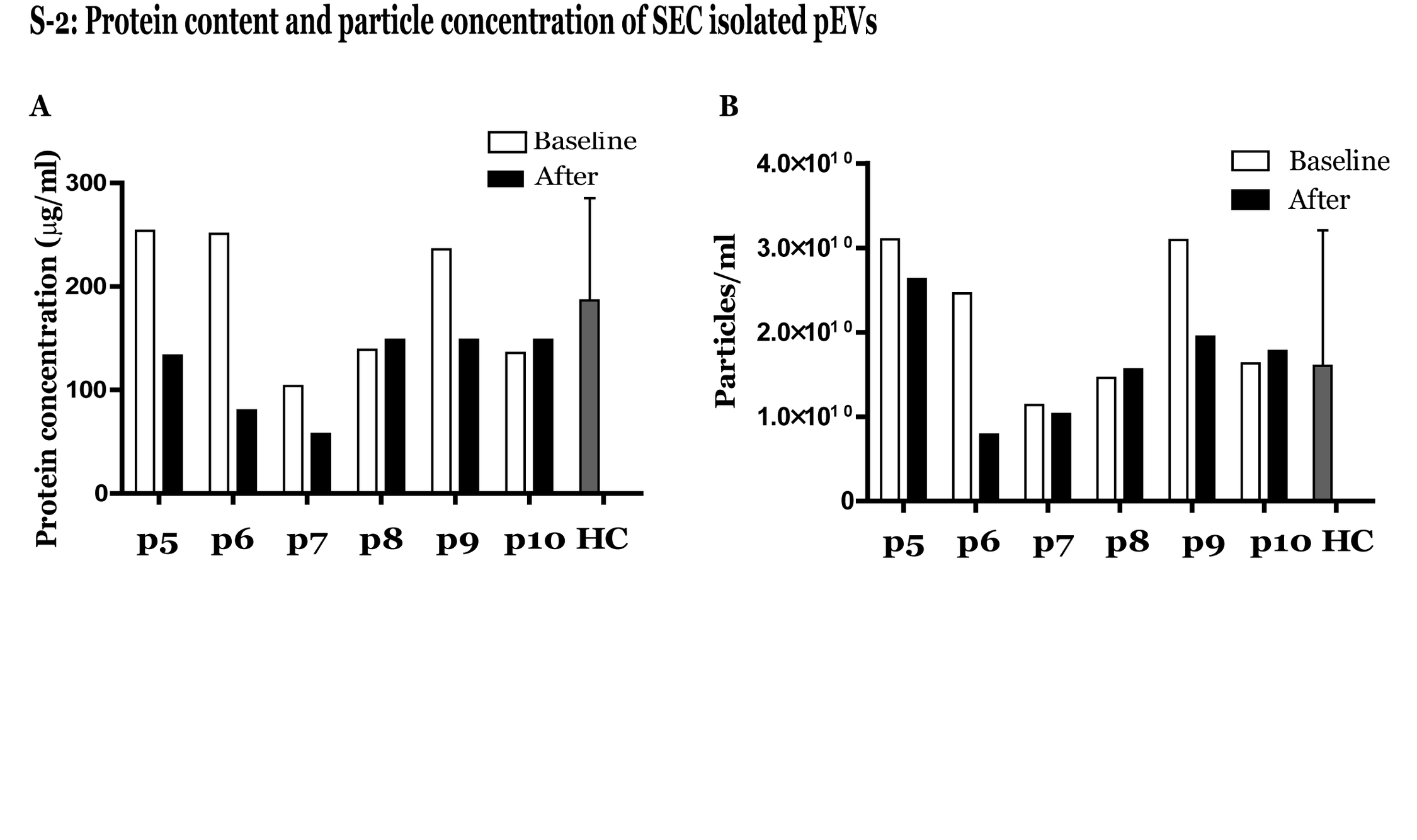

Supplement: S2 Fig — Protein content and particle concentration of pEVs isolated from 6 RA patients before (white bars) and after (black bars) etanercept treatment and 5 age matched healthy controls (grey bar) detected by resp. micro-BCA (A) and NTA (B). (TIF) [file pone.0236508.s002.tif]

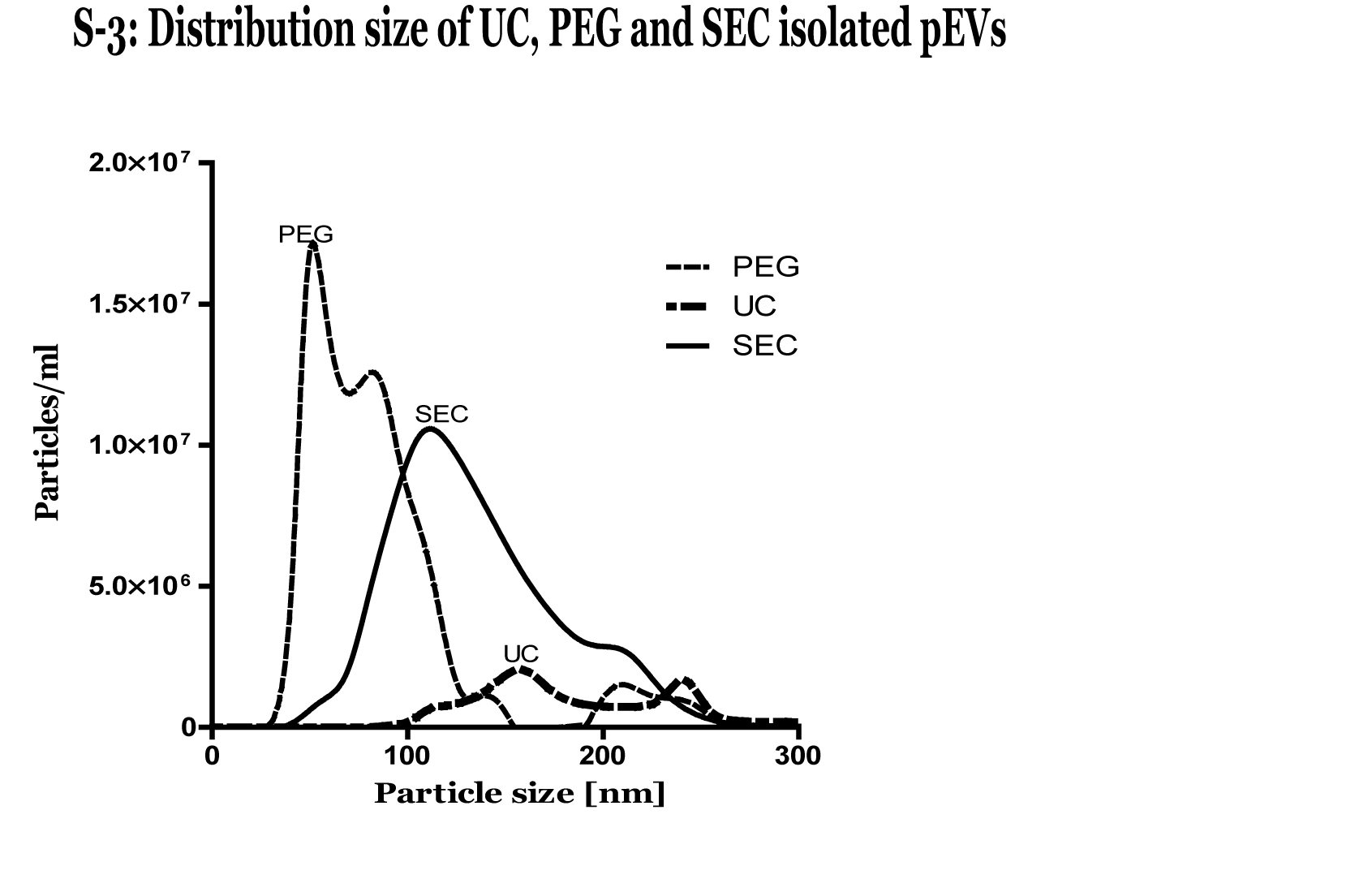

Supplement: S3 Fig — Distribution size of pEVs isolated by UC, PEG or SEC from the same RA patient. Most monodisperse particles were observed after SEC isolation. (TIF) [file pone.0236508.s003.tif]

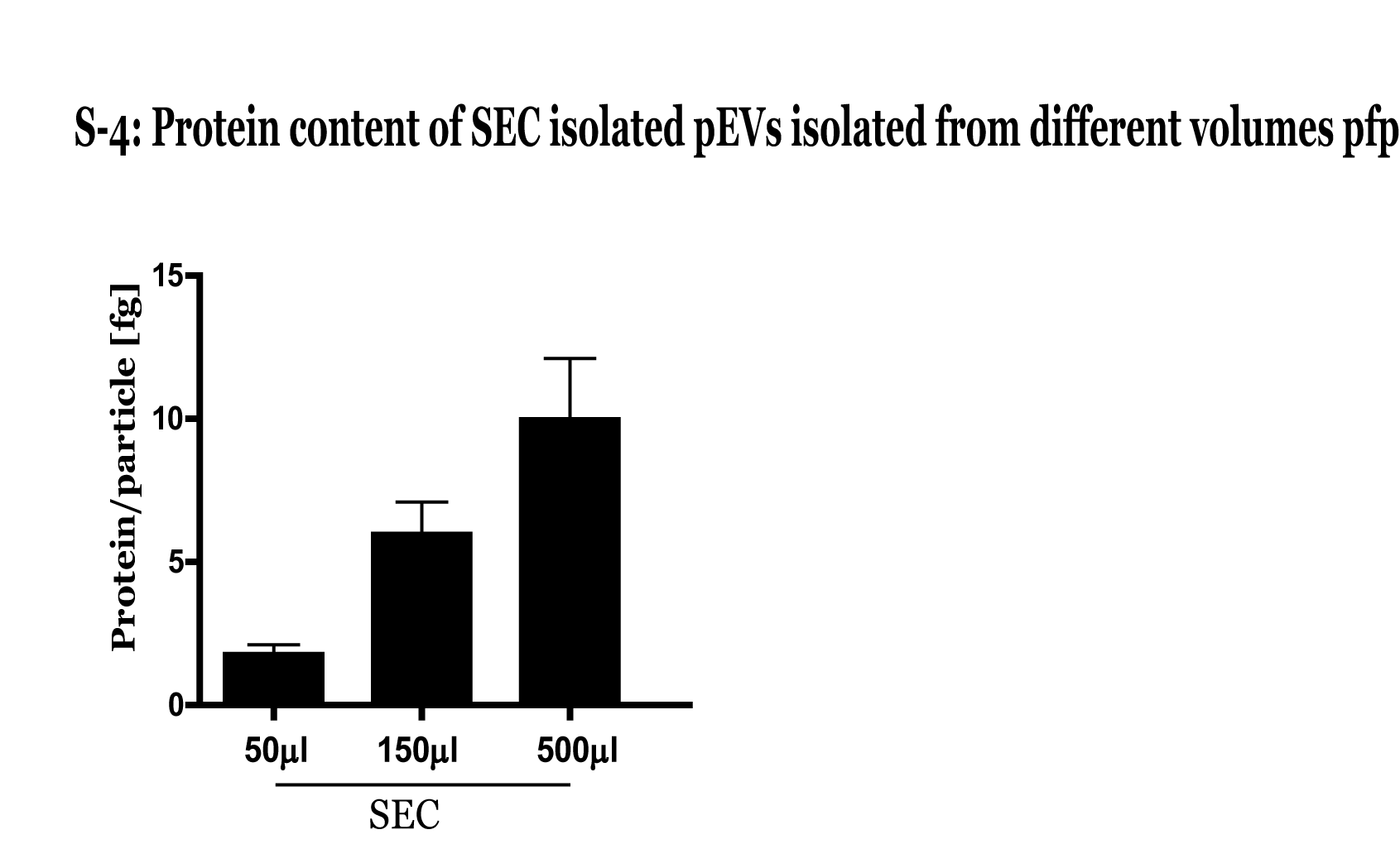

Supplement: S4 Fig — Protein content per particle of pEVs isolated by SEC of different pfp volumes (50-150-500μl) suplemented with pbs to 500μl. Enhanced protein per particle levels were observed with higher pfp volumes. (TIF) [file pone.0236508.s004.tif]

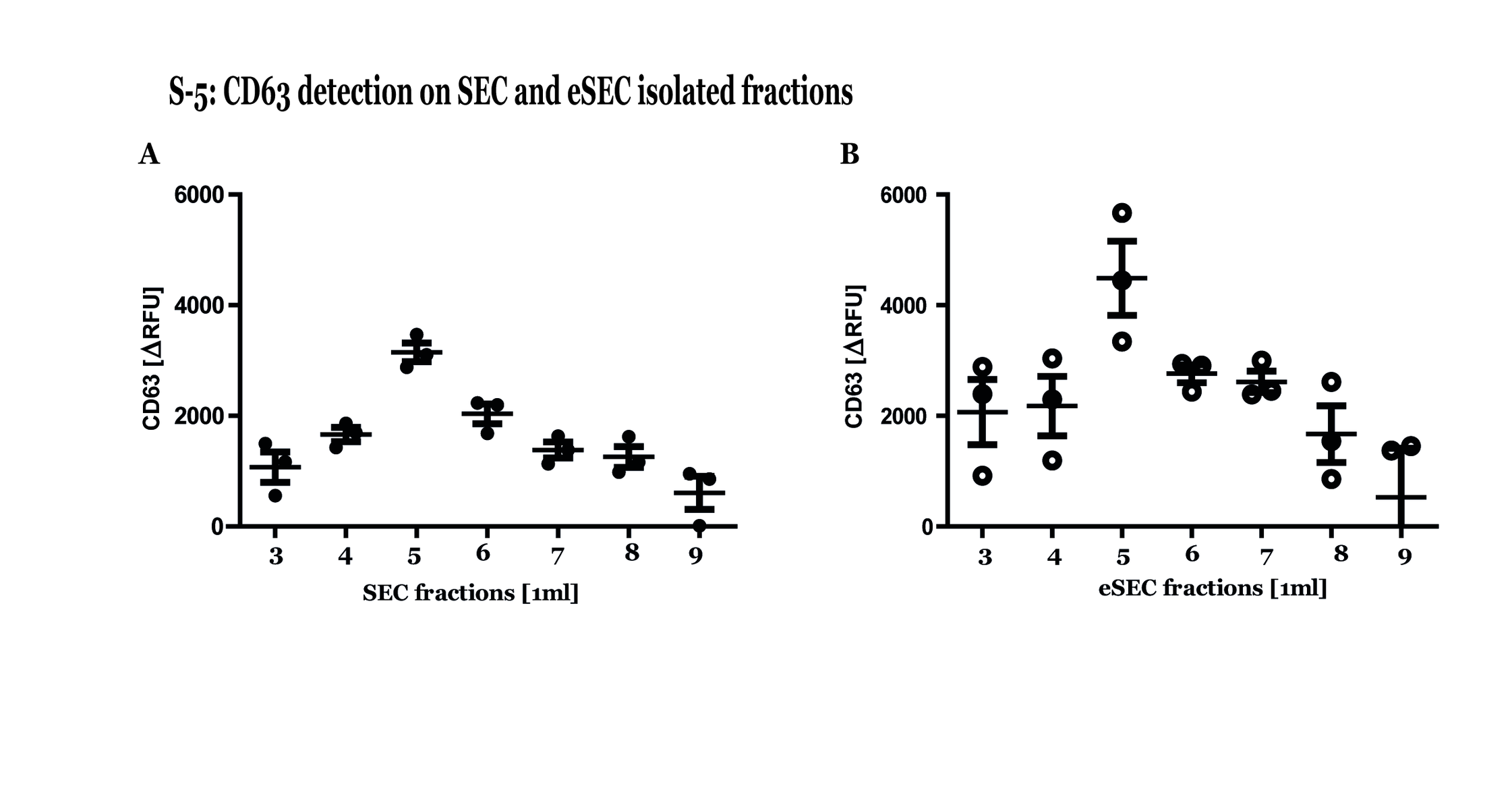

Supplement: S5 Fig — CD63 was detected in different SEC and eSEC isolated fractions. Highest CD63 content was observed in the pEV fraction (fraction 5). (TIF) [file pone.0236508.s005.tif]

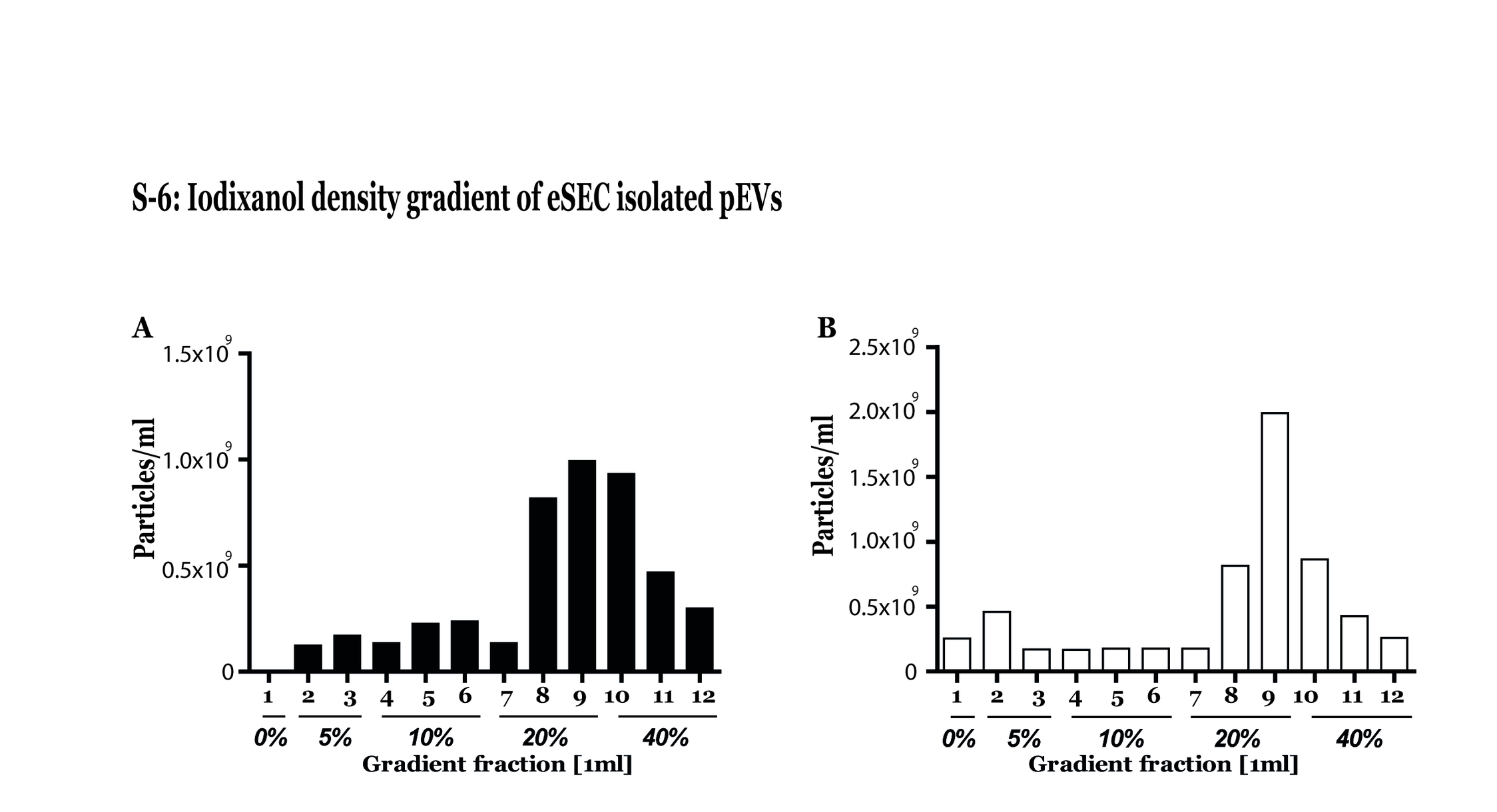

Supplement: S6 Fig — To detemined the density of the SEC and eSEC isolate pEVs a discontinuous iodixanol gradient was used and the most pEVs were detected in the 20% density layer. (TIF) [file pone.0236508.s006.tif]

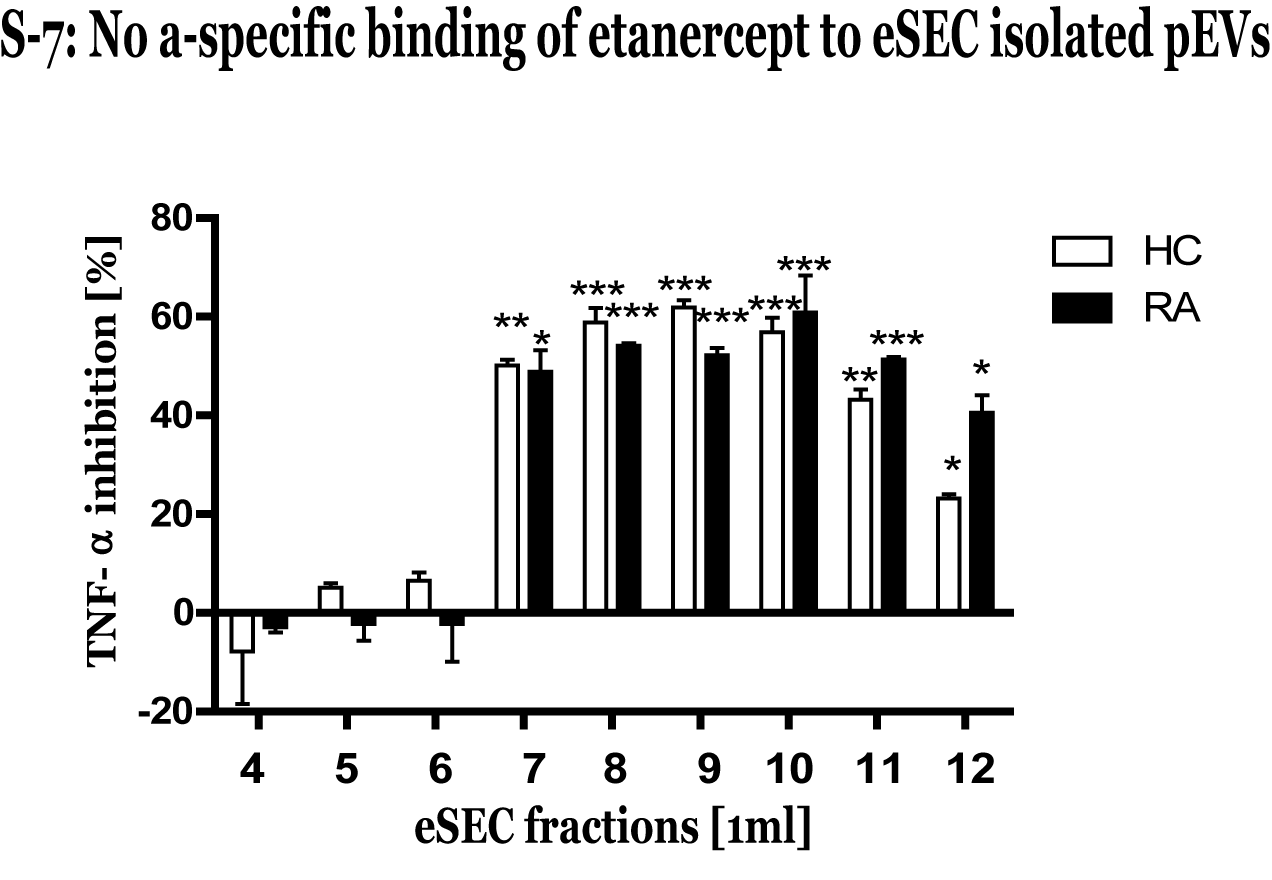

Supplement: S7 Fig — Detemination of TNF-α inhibition in eSEC isolated fractions of HC- or RA- pEVs (isolated by eSEC) preincubated with etanercept (1 μg/ml. No TNF-α inhibition was observed in eSEC fractions 4, 5 and 6. (TIF) [file pone.0236508.s007.tif]
