# Supplementary material for: An optimized method for plasma extracellular vesicles isolation to exclude the copresence of biological drugs and plasma proteins which impairs their biological characterization
Source: PLoS One. 2020 Jul 29;15(7):e0236508. doi: 10.1371/journal.pone.0236508 (PMC7390383; doi:10.1371/journal.pone.0236508)
Supplement: S1 Data — (DOCX) [file pone.0236508.s008.docx]

**Supplementary data.**

**Material and Methods**

**CD63 detection**

CD63 was detected by an in house developed ELISA. In short, 50µl SEC or eSEC fractions were coated overnight to a 96 wells high binding white flat bottom plate (Greiner Bio-One, Frickenhausen, Germany) by RT, After 3 times washing with PBS-Tween 50µl 0,1ug/ml mouse-anti-humCD63-PE (Biolegend, London, United Kingdom) was added and after 2hr incubation by RT plate was washed 3 times and PE was measured in a fluorescence meter (Clariostar. BMG) as an amount of CD63.

**Iodixanol density gradient**

The density of the pEV samples was determined by using an Optiprep^TM^ density gradient. Briefly, a discontinuous iodixanol gradient was prepared by diluting a stock solution of Optiprep^TM^ (60% w/v) (Sigma-Aldrich, St. Louis, MO, USA) with 0.25 M sucrose/10 mM Tris, pH 7.5 to generate 40%, 20%, 10% and 5% w/v iodixanol solutions. With care, the discontinuous iodixanol gradient was generated by sequentially layering 3 ml each of 40, 20 and 10% (w/v) iodixanol solutions, followed by 2.5 ml of 5% iodixanol solutions in 14x95mm Polypropylene centrifuge tubes (Beckman Coulter, Woerden, The Netherlands). 500µl pEV sample was overlaid in the discontinuous iodixanol gradient and centrifuged using SW40 Ti rotor for 16 hours at 100,000g by 4^0^C. Fractions of 1 ml were collected from the top of the gradient. To each fractions 11 ml PBS was added and after centrifugation for 2 hours by 110,000g particles in pellet were measured in optimal dilutions by NTA.
